# Frequency and significance of the novel single nucleotide missense polymorphism Val109Asp in the human gene encoding omentin in Caucasian patients with type 2 diabetes mellitus or chronic inflammatory bowel diseases

**DOI:** 10.1186/1475-2840-6-3

**Published:** 2007-02-13

**Authors:** Andreas Schäffler, Martina Zeitoun, Hella Wobser, Christa Buechler, Charalampos Aslanidis, Hans Herfarth

**Affiliations:** 1 Department of Internal Medicine I, Regensburg University Medical Center, Germany; 2 Institute of Clinical Chemistry and Laboratory Medicine, Regensburg University Medical Center, Germany; 3 Department of Medicine, Division of Gastroenterology and Hepatology, University of North Carolina, Chapel Hill, NC, USA

## Abstract

**Background:**

The omental adipose tissue is pathogenetically involved in both type 2 diabetes mellitus (T2D) and chronic inflammatory bowel diseases (IBD) such as Ulcerative colitis (UC) and Crohn's Disease (CD). Thus, adipokines secreted from omental adipose tissue might play an important role in these diseases. Omentin represents a new adipokine expressed in and secreted by omental adipose tissue. Therefore, it was the aim to investigate the putative role of a newly described sequence missense variation in the human omentin gene.

**Methods:**

The Val109Asp single nucleotide miss-sense polymorphism and the His86His polymorphism in exon-4 of the omentin gene were newly identified by random sequencing. Only the miss-sense polymorphism was investigated further. Genotyping was performed by restriction fragment length polymorphism (RFLP) analysis of amplified DNA fragments. Three different cohorts of well-characterized individuals were included in the study. 114 patients suffering from T2D, 190 patients suffering from IBD (128 with CD and 62 with UC) and 276 non-diabetic healthy controls without any history for IBD were analyzed.

**Results:**

The following allelic frequencies were determined: controls: Val-allele: 0.26, Asp-allele: 0.74; T2D: Val-allele: 0.3, Asp-allele: 0.7; IBD: Val-allel: 0.31, Asp-allele: 0.69. UC and CD patients did not differ in regard to the allelic frequency. Similarly, controls, T2D patients and IBD patients did not show significant differences in genotype distribution among each other. Disease manifestation and pattern of infestation were not related to genotype subgroups, neither in CD nor in UC. Furthermore, there was no significant association between genotype subgroups and anthropometric or laboratory parameters in T2D patients.

**Conclusion:**

Based on sequence comparisons and homology searches, the amino acid position 109 is conserved in the omentin gene of humans, mice and chimpanzee but is not completely conserved between other omentin homologous genes. Moreover, position 109 lies outside the fibrinogen domain. Due to these structural features and based on the present data, the Val109Asp sequence variation is more a single nucleotide polymorphism than a real disease-causing mutation.

## Background

Adipokines [1] such as leptin, resistin and adiponectin are currently investigated as potential future drug targets in type 2 diabetes mellitus (T2D), lipid metabolism, endothelial dysfunction and inflammatory diseases in general. Therefore, the discovery of yet unknown adipokines represents a main target in the field of metabolic and immunological basic research.

In 2003, a new cDNA [2] named "omentin" (gene bank accession number: AY549722) was described and reported to be expressed specifically in human omental adipose tissue. An abstract published in *Diabetes *in the year 2003 [3] reported omentin as a new adipokine secreted from omental adipose tissue. In 2005, the genomic structure, promoter sequence, exon-intron organization, predicted amino acid sequence and putative protein structure and differential expression of omentin in omental adipose tissue samples obtained from patients with chronic inflammatory bowel diseases was published by our group [4]. Based on the predicted amino acid sequence, we could demonstrate a 100 % homology between the omentin amino acid sequence and a protein termed intelectin [4]. Human intelectin was characterized as a novel soluble lectin that recognizes galactofuranose in carbohydrate chains of bacterial cell walls [5]. In 2006, Yang et al. demonstrated that omentin is capable of enhancing insulin-mediated glucose-uptake in adipocytes [6]. Furthermore, they found that omentin is predominantly expressed in visceral but not in subcutaneous adipose tissue, however, with adipose tissue stromal cells being the main source of omentin [6]. Omentin was also detectable in human serum by Western blot analysis and recombinant omentin in vitro did not affect basal but enhanced insulin-stimulated glucose uptake in human adipocytes. Although the mechanism is far from clear and no specific omentin receptor has been described so far, omentin seems to induce adipocytic Akt (protein kinase) phosphorylation [6].

Omentin might exert both metabolic and immuno-modulatory effects. Since T2D is associated with visceral adipose tissue hypertrophy and since this disease has been regarded as a chronic and low grade state of inflammation, it seems reasonable to investigate cohorts of patients suffering from T2D for sequence variations within the omentin gene. Additionally, omental adipose tissue is involved in the transmural and intra-abdominal inflammatory process observed in Crohn's Disease (CD) [7-9]. These adipose tissue changes in patients with CD are cited as *fat hypertrophy*, *fat wrapping *(fat creeping upon the bowel) or *creeping fat *and have long been recognized by surgeons as a phenomenon suitable for delineating the extent of active disease [7,8]. The identification of a distinct secretion pattern of adipokines from *creeping fat *in CD [10-14] and from mesenteric adipose tissue in chronic inflammatory bowel diseases (IBD) can be momentarily considered as work in progress. The characterization of omental adipose tissue by its highly active secretory products might lead to the discovery of specific discrimination and activity markers in IBD and might provide future targets for drug therapy. Based on this, it seems also reasonable to investigate omentin sequence variations in the context of chronic inflammatory bowel diseases (IBD) such as CD and Ulcerative Colitis (UC).

Therefore, it was our aim

- to search for yet unknown missense sequence variations within the coding sequence of the human omentin gene,

- to determine the allelic frequency of newly discovered missense single nucleotide polymorphisms in a cohort of Caucasian patients suffering from T2D and IBD,

- to describe possible associations of genotype subgroups with standard metabolic parameters and anthropometric data in T2D and disease manifestation in IBD.

## Methods

### Study populations

As healthy controls, 276 non-diabetic subjects (110 males and 166 females) without any history of chronic inflammatory bowel diseases were included in the study. Blood (EDTA-whole blood and serum) was drawn after an overnight fast. All participating individuals were informed about the aim of the study and gave informed consent. The study was approved by the local ethical committee. The characteristics of the control subjects are summarized in table 1.

**Table 1:** Characteristics of 276 non-diabetic control subjects.

	**Total**	**Males**	**Females**	**p**
**n **(%)	**276 **(100)	**110 **(39.9)	**166 **(60.1)	
**Age **(years ± SEM)	38.9 ± 0.9	38.5 ± 1.5	39 ± 1.0	n.s.
**BMI **(kg/m^2 ^± SEM)	27.9 ± 0.4	27.8 ± 0.6	28.1 ± 0.6	n.s.
**Cholesterol, total **(mg/dl ± SEM)	221 ± 3	220 ± 5	223 ± 4	n.s.
**HDL-cholesterol **(mg/dl ± SEM)	58 ± 1	50 ± 2	62 ± 2	p < 0.0001
**LDL-cholesterol **(mg/dl ± SEM)	133 ± 3	130 ± 5	134 ± 3	n.s.
**Triglycerides **(mg/dl ± SEM)	168 ± 15	199 ± 23	151 ± 21	p = 0.03
**Uric acid **(mg/dl ± SEM)	5.7 ± 0.1	6.8 ± 0.1	4.9 ± 0.1	p < 0.0001

SEM = standard error of the mean, n.s. = not significant

114 patients suffering from a known history of T2D and 190 patients suffering from IBD such as UC or CD were referred from the endocrinological or gastroenterological outpatient clinic, respectively (Department of Internal Medicine I, University Hospital of Regensburg, Germany). In contrast to CD, UC is not associated with adipose tissue changes and thus is suitable as a control. The characteristics of the diabetic study cohort (114 patients) are summarized in table 2, the medication is given in table 3. The characteristics of the patients suffering from chronic inflammatory bowel diseases are summarized in table 4.

**Table 2:** Characteristics of 114 patients with diabetes mellitus type 2

	**Total**	**Males**	**Females**	**p**
**n **(%)	**114 **(100)	**68 **(59.6)	**46 **(40.4)	
**Age **(years ± SEM)	62.9 ± 0.9	60.8 ± 1.3	66.2 ± 1.4	* 0.008
**BMI **(kg/m^2 ^± SEM)	28.8 ± 0.7	30.3 ± 0.9	26.7 ± 0.9	* 0.016
**Diabetes duration **(years ± SEM)	7.8 ± 0.8	7.6 ± 1.0	8.1 ± 1.4	ns
**HbA_1c _**(% ± SEM)	7.5 ± 0.2	7.6 ± 0.2	7.4 ± 0.3	ns
**Cholesterol, total **(mg/dl ± SEM)	199 ± 5	196 ± 7	203 ± 7	ns
**HDL-cholesterol **(mg/dl ± SEM)	49 ± 2	46 ± 2	54 ± 3	* 0.03
**LDL-cholesterol **(mg/dl ± SEM)	117 ± 5	122 ± 7	110 ± 6	ns
**Triglycerides **(mg/dl ± SEM)	176 ± 11	188 ± 14	158 ± 15	ns
**Uric acid **(mg/dl ± SEM)	6.7 ± 0.6	7.3 ± 0.9	5.9 ± 0.4	ns

SEM = standard error of the mean, ns = not significant, * = statistical significance between males and females

**Table 3:** Medication in 114 patients with type 2 diabetes mellitus.

**Therapy**	**Diabetes**	**Hypertension**	**Lipid-lowering**
	patients n (%) **114 (100)**	patients n (%) **88 (77.2)**	patients n (%) **28 (24.6)**

**Medications**			
diet	114 (100)		
sulfonylureas	29 (25.4)		
metformin	44 (38.6)		
glitazones	2 (1.8)		
glinides	7 (6.1)		
carboanhydrase inhibitors	2 (1.8)		
insulin (alone/combined)	52 (45.6)		
statins/fibric acid			28 (24.6)
ACE inhibitors		40 (35.1)	
calcium-channel blockers		19 (16.7)	
diuretic drugs		41 (36.0)	
beta-blockers		32 (28.1)	
others		3 (2.6)	

Since combined therapies are possible, percentages/numbers might exceed 100

**Table 4:** Study cohort of 190 patients suffering from chronic inflammatory bowel diseases.

	**Total population**	**Crohn's Disease**	**Ulcerative Colitis**
**n (%)**	190 (100%)	128 (67.4%)	62 (32.6%)
**age (years) **± SEM	37 ± 10	36 ± 10	39 ± 10
**female n (%)**	85 (44.7%)	64 (50.0%)	21 (34%)
**male n (%)**	106 (55.3%)	64 (50.0%)	42 (66%)
**Body mass index (BMI) kg/m^2 ^**± SEM	23.8 ± 3.5	23.3 ± 3.6	24.7 ± 3.2
**C-reactive protein mg/dl **± SEM	14.6 ± 13.5	15.1 ± 13.0	13.9 ± 14.4
**systemic steroids n (%)**	79 (41%)	46 (36%)	33 (53%)
**topical steroids n (%)**	47 (25%)	24 (18%)	23 (37%)
**other immuno-suppressants n (%)**	48 (25%)	32 (24%)	16 (25%)
***Vienna classification [22] *n (%): *L1***	-	21 (16.4%)	-
***L2***	-	24 (18.8%)	-
***L3***	-	67 (52.3%)	-
***L4***	-	16 (12.5%)	-
***B1***	-	35 (27.3%)	-
***B2***	-	35 (27.3%)	-
***B3***	-	58 (45.4%)	-

Vienna classification: L1 = terminal ileum, L2 = colon, L3 = ilecolon, L4 = upper gastrointestinal tract; B1 = non-stricturin, non-penetrating, B2 = stricturing, B3 = penetrating

### Identification of two new single nucleotide polymorphisms

Recently, we established exon-specific genomic PCR amplifications for the 8 exons of the human omentin gene [3]. Using the random sequencing approach of genomic DNA obtained from healthy individuals, we could identify two common and yet unpublished single nucleotide missense polymorphism (SNP). In exon-4, the nucleotide +326 (numbering relatively to the ATG start codon) is polymorphic (A/T). Thus, the codon GAC is replaced by GTC changing the amino acid Asp to Val at position 109. The second polymorphism identified (His86His) does not change the amino acid and is located at nucleotide 258 (C/T) within exon-4. This latter SNP was therefore not investigated further.

### PCR-based RFLP analysis of the omentin Val109Asp SNP

**Figure 1:**
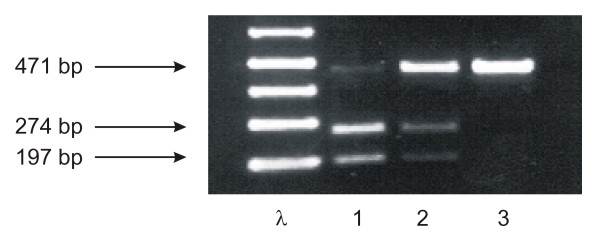
**Genomic PCR-based RFLP analysis of the Val109Asp SNP**. λ = DNA molecular weight marker, 1 = Val/Val homozygotes, 2 = Asp/Val heterozygotes, 3 = Asp/Asp homozygotes.

In order to investigate, whether this sequence variation is a real polymorphism according to the Hardy-Weinberg equilibrium, a PCR-based RFLP (restriction fragment length polymorphism) analysis was established for a simple and inexpensive testing of larger cohorts of patients. Genomic DNA was prepared from whole venous blood using a commercially available DNA isolation kit (Qiagen, Hilden, Germany). A 471 bp DNA fragment was amplified under standard conditions by genomic PCR (annealing temperature: 58°C) in a GeneAmp9600^R ^thermal cycler (Perkin Elmer) using the upstream primer 5'-GAGCCTTTAGGCCATGTCTCT-3' and the downstream primer 5'-CTCTCCTTCTTCTCCAGCCCAT-3'. The PCR product was then digested at 37°C by the restriction enzyme AccI (Roche Mannheim, Germany) and separated by 3% agarose gel electrophoresis. Within exon-4, the polymorphic codon GTC encoding Val is part of a *AccI *recognition site, whereas the codon GAC eliminates the AccI recognition site. Thus, in RFLP agarose gel electrophoresis (fig. 1), Val/Val homozygotes show two bands of 274 bp and 197 bp, Val/Asp heterozygotes show three bands of 471 bp, 274 bp and 197 bp, and Asp/Asp homozygotes show one single band of 471 bp (fig. 1).

### Statistics

For statistical analysis, the SPSS/PC+ statistical software package was used (SPSS 12.0). Subjects were compared for differences in anthropometric and biochemical data according to their genotypes by two tailed Mann-Whitney or Kruskal-Wallis tests for comparison of two or more independent samples. For correlation analysis, two tailed Spearman test was used as well as Pearsons χ^2 ^test for associations between classified variables. Data are expressed as means and standard error of the mean (SEM) for simplicity. A p value below 0.05 (two tailed) was considered to be statistically significant. Adjustments according to Bonferroni were not done.

## Results

### Characteristics of the study population

#### Cohort of non-diabetic controls without a history of IBD

276 non-diabetic control subjects (110 males and 166 females) were included. Table 1 shows the characteristics of the control subjects. Individuals were comparable with respect to age and BMI. As a well-known metabolic feature, females had significantly higher HDL values (p < 0.0001), lower triglyceride levels (p = 0.03) and lower levels of uric acid (p < 0.0001) than males. All calculations were repeated with a completely balanced gender relation, but the results presented in the manuscript were not influenced when data were compared either to the total control group or to a completely gender-balanced control group.

#### Cohort of patients suffering from T2D

In total, 114 patients (68 males, 46 females) with a known history of T2D were included in the study. The mean age was 62.9 ± 0.9 years, mean BMI was 28.8 ± 0.7 kg/m^2 ^and mean diabetes duration was 7.8 ± 0.8 years. The detailed characteristics of the entire study population are summarized in table 2. Both genders were comparable with respect to mean diabetes duration, medication, mean HbA1c levels, total cholesterol, LDL-cholesterol, HDL-cholesterol, triglycerides, uric acid and creatinine. Females were significantly older (66.2 ± 1.4 vs. 60.8 ± 1.3 years, p = 0.008) and had a lower BMI (26.7 ± 0.9 vs. 30.3 ± 0.9 kg/m^2^, p = 0.016) and higher HDL-cholesterol levels (54 ± 3 vs. 46 ± 2 mg/dl) than males (table 2).

Age was positively associated with BMI (p = 0.003), presence of peripheral arterial occlusive disease (p = 0.041), CRP levels (p = 0.001), and negatively associated with creatinine clearance (p < 0.0001). Diabetes duration was positively associated with the presence of peripheral neuropathy (p < 0.0001), retinopathy (p = 0.001) and coronary artery disease (p = 0.024). BMI was positively associated with total cholesterol (p = 0.032), triglycerides (p = 0.002), LDL-cholesterol (p = 0.013), and negatively associated with HDL-cholesterol (p = 0.003). Based on these well known correlations (data not shown), the present study cohort represents the typical characteristics of a type 2 diabetic population. The medication of the patients is shown in table 3.

#### Cohort of patients suffering from UC or CD

In total, 190 patients (85 females, 106 males) with IBD were included, 128 suffering from CD and 62 suffering from UC. The mean BMI was 23.8 ± 3.5 kg/m^2 ^and the mean CRP level was 14.6 ± 13.5 mg/dl. 41 % of the total study population were treated with systemic steroids, 25 % with topical steroids and 25 % with other immunosuppressants. The detailed study characteristics and the Vienna classification of patients suffering from Crohn's disease are given in table 4.

### Genotyping and allelic frequency of the Asp109Val SNP within exon-4

#### Cohort of non-diabetic controls without a history of IBD

Out of 276 control subjects, 14 (5.1 %) were homozygous for the Val/Val genotype, 124 (44.9 %) were heterozygous for the Val/Asp genotype and 138 (50.0 %) were homozygous for the Asp/Asp genotype. According to the Hardy-Weinberg equilibrium, allelic frequency was calculated to be 0.26 for the Val allele and 0.74 for the Asp allele. Anthropometric and measured laboratory parameters did not differ significantly between genotype subgroups.

#### Cohort of patients suffering from T2D

Genotyping was successfully performed in all of the 114 patients with T2D (table 5). Out of 114 patients, 12 (10.5 %) were homozygous for the Val/Val genotype, 44 (38.6 %) were heterozygous for the Val/Asp genotype and 58 (50.9 %) were homozygous for the Asp/Asp genotype. According to the Hardy-Weinberg equilibrium, allelic frequency was calculated to be 0.3 for the Val allele and 0.7 for the Asp allele. Concerning lipid, laboratory and anthropometric parameters, no significant differences were found between the three genotype subgroups. Moreover, when combining Val/Asp with Asp/Asp genotypes or Val/Val with Val/Asp genotypes as one single group, no significant differences could be found concerning the clinical and laboratory parameters.

**Table 5:** Anthropometric parameters and laboratory parameters in genotype subgroups of the omentin Val109Asp SNP (114 patients with type 2 diabetes mellitus

	**Val/Val**	**Val/Asp**	**Asp/Asp**	**p**
**n (%)**	12 (10.5)	44 (38.6)	58 (50.9)	n.s.
**Age **(years) ± SEM	61.2 ± 3	62.7 ± 1.5	63.5 ± 1.5	n.s.
**BMI **(kg/m^2^) ± SEM	27.9 ± 1.4	28.1 ± 0.9	29.6 ± 1.1	n.s.
**HbA1c **(% ± SEM)	7.5 ± 0.4	7.4 ± 0.3	7.7 ± 0.3	n.s.
**Cholesterol **(mg/dl ± SEM)	197 ± 14	193 ± 9	203 ± 7	n.s.
**HDL-cholesterol **(mg/dl ± SEM)	45 ± 3	50 ± 3	50 ± 2	n.s.
**LDL-cholesterol **(mg/dl ± SEM)	122 ± 17	121 ± 8	111 ± 6	n.s.
**Triglycerides **(mg/dl ± SEM)	216 ± 40	167 ± 18	172 ± 12	n.s.

mean ± SEM, n.s.= not significant

#### Cohort of patients suffering from UC or CD

All 190 patients suffering from IBD were successfully genotyped. Out of these, 17 (8.9 %), were homozygous for the Val/Val genotype, 82 (43.2 %) were heterozygous for the Val/Asp genotype and 91 (47.9 %) were homozygous for the Asp/Asp allele. Accordingly, allelic frequency was calculated to be 0.31 for the Val allele and 0.69 for the Asp allele. Genotype distribution of subgroups of patients with UC and CD are summarized in table 6. No significant differences in the genotype distribution and allelic frequencies were found between both subgroups (table 6) and when compared to control subjects and patients suffering from T2D (data not shown).

**Table 6:** Genotype distribution of the Val109Asp SNP in 190 patients suffering from chronic inflammatory bowel diseases

	**Total population (n = 190)**	**Crohn's Disease (n = 131)**	**Ulcerative Colitis (n = 59)**	**p**
**Genotype**				
Val/Val n (%)	17 (8.9)	11 (8.4)	6 (10.1)	n.s.
Val/Asp n (%)	82 (43.2)	57 (43.5)	25 (42.4)	n.s.
Asp/Asp n (%)	91 (47.9)	63 (48.1)	28 (47.5)	n.s.

n.s. not significant between groups

## Discussion

There are several considerations why it seems reasonable to investigate omentin sequence variations with respect to IBD such as UC and CD. First of all, there is increasing evidence showing that omental adipose tissue is actively involved in the pathogenesis of CD [7,8,12,15]. Transmural adipose tissue inflammation, commonly cited as "creeping fat" and omental adipose tissue hypertrophy are oftenly present in active CD and are associated with the systemic inflammatory response as measured by C-reactive protein [16]. Moreover, visceral adipose tissue in patients suffering from CD does secret significantly higher amounts of adiponectin, macrophage colony stimulating factor (MCSF), monocyte chemoattractant protein-1 (MCP-1), leptin, and migration inhibitory factor (MIF) than patients suffering from UC [12]. Most interestingly, we could demonstrate that omental adipose tissue obtained from patients suffering from CD do express varying degrees of omentin mRNA [4]. Since omentin and intelectin were reported to be identical human proteins [4], it is important to emphasize that intelectin was earlier shown to represent a new type lectin recognizing galactofuranose residues in bacterial cell walls. When combining the intestinal expression profile of omentin (small and large intestine, intestinal adipose tissue) with the functional data on the recognition of bacteria-specific components in the host, omentin could be involved in intestinal defense mechanisms in CD. Especially the intestinal adipose tissue has been discussed as a primary barrier against bacterial translocation in IBD [7,8]. In the present study we could not find a significantly different allelic frequency of the Val109Asp SNP in patients suffering from IBD when compared to healthy controls or T2D patients. Moreover, we could not demonstrate any associaton of genotypes with disease manifestation or clinical parameters in these patients. However, these results do not exclude the putative role of yet unknown omentin sequence variations in the pathogenesis of IBD and the descriptive data on omentin expression and function do encourage to investigate this further.

There are several considerations why it seems reasonable to investigate omentin sequence variations with respect to type 2 diabetes mellitus. Omental adiposity [17,18] preceds the development of insulin resistance and T2D and is one of the major components of the metabolic syndrome. Since adipokines derived from omental adipose tissue are clearly involved in insulin resistance, dyslipidemia and coronary artery disease [1,19-21], new adipokines such as omentin might play an important role in the pathogenesis of T2D and associated metabolic disorders. However, in the present study we failed to demonstrate a significantly different allelic frequency of the Val109Asp SNP in patients with T2D when compared to healthy controls or patients suffering from IBD. Moreover, we could not find any associations of the SNP with standard anthropometric and metabolic parameters. However, these results do not exclude the putative role of yet unknown omentin sequence variations in the pathogenesis of T2D.

Since the allelic frequency of the Val109Asp SNP does not differ between healthy controls, T2D patients and patients suffering from IBD, the amino acid position at 109 might not be critical for the function of the omentin protein. However, unless functional studies with site directed mutagenesis have been performed, it is not possible to speculate about the functional consequence.

Affinity chromatography using galactose-sepharose and protein analysis by SDS-PAGE revealed that omentin forms a 120 kDa homotrimer linked by disulfide bonds [5]. Since omentin lacks a classical transmembrane domaine [4,5] and is detectable in human sera, the protein seems to be a secreted protein. Human omentin contains two potential N-glycosylation sites at Asn154 and at Asn163 [5], but not at the Val/Asp109 site investigated in this study (additionally, Val/Asn can not form disulfide binding sites or a phosphorylation sites). The Val/Asp109 site lays carboxyterminally outside of a fibrinogen domain spanning from amino acid 38 to amino acid 82. Amino acid position 109 is conserved between humans, mice and chimpanzee. However, when compared to the homologous proteins XCGL (*Xenopus laevis *cortical granule lectin), LSL (*L. japonica *lamprey serum lectin) and αGSL (*H. roretzi *ascidian glactose-specific lectin), the site at position 109 does not represent a completely conserved consensus amino acid position [5], whereas the direct adjacent site at 108 contains a highly conserved amino acid (alanine). Although there do not exist experimental data on the functional consequence of the Val109Asp mutation on protein function, the proximity of the mutated amino acid to the higly conserved amino acid at site 108 might be of functional relevance.

Since we decided not to investigate the allelilc frequency of the His86His SNP in our cohort, we cannot exclude that this SNP might stand in a putative linkage dysequilibrium with other gene polymorphisms playing a role in metabolism. Future studies might address this question.

## Conclusion

Based on sequence comparisons, homology searches and considerations concerning the protein domain structure, there might be an explanation why the Val109Asp SNP does not associate with the clinical parameters investigated in our cohort of patients and why allelic frequencies are very similar between subgroups. However, unless functional studies have been performed, it is not possible to speculate about the functional consequence of the mutation. Based on expressional and functional considerations, additional omentin sequence variations not yet described should be investigated in cohorts of patients with T2D or IBD.

## Authors' contributions

AS and MZ carried out the molecular studies and measured the laboratory parameters. CB participated in the study design and coordinated and helped to draft the manuscript. HH, AS, HW and MZ participated in collecting the blood samples and in building up the study cohorts.

## Declaration of competing interests

The author(s) declare that they have no competing interests.
